# Develop a prognostic and drug therapy efficacy prediction model for hepatocellular carcinoma based on telomere maintenance-associated genes

**DOI:** 10.3389/fonc.2025.1544173

**Published:** 2025-02-14

**Authors:** Jian-Hao Zheng, Ding Shi, Yun-Jie Chen, Jian-Ping Liu, Zheng Zhou

**Affiliations:** ^1^ Department of Gastroenterology, Ningbo No.2 Hospital, Ningbo, China; ^2^ Department of General Surgery, Ningbo No.2 Hospital, Ningbo, China

**Keywords:** hepatocellular carcinoma, telomere maintenance genes, risk model, nomogram, immune evasion

## Abstract

**Background:**

Hepatocellular carcinoma (HCC) poses a substantial global health challenge because of its grim prognosis and limited therapeutic options. Telomere maintenance mechanisms (TMM) significantly influence cancer progression, yet their prognostic value in HCC remains largely unexamined. This research aims to establish a telomere maintenance-associated genes(TMGs)-based prognostic model using transcriptomic and clinical data to evaluate its effectiveness in predicting patient outcomes in HCC.

**Methods:**

The identified differentially expressed genes (DEGs) were derived from the analysis of transcriptomic and clinical information sourced from the database of the Cancer Genome Atlas (TCGA) and were cross-referenced with TMGs. Candidate risk factors were initially assessed using univariate Cox regression, subsequently followed by LASSO, and then refined through multivariate Cox regression to establish a risk prediction model. This model’s predictive accuracy was validated through Kaplan-Meier(K-M) survival analysis, with external validation in the Gene Expression Omnibus (GEO) dataset. Additionally, a nomogram incorporating age and tumor stage was developed. Tumor mutation burden (TMB), immune profile, and drug sensitivity in HCC were also analyzed. Furthermore, we employed RT-PCR to confirm the expression levels of the genes related to TMGs in HepG2 cell lines.

**Results:**

A prognostic model comprising 3 core genes was constructed, with high-risk individuals showing significantly lower overall survival (OS). The association between elevated TMB and diminished survival in high-risk patients was uncovered through TMB analysis. Immune profiling indicated notable disparities in immune infiltration among these groups, with high-risk patients displaying elevated Tumor Immune Dysfunction and Exclusion (TIDE) scores, suggesting potential immune evasion.

**Conclusion:**

In short, our prognosis model based on TMGs effectively categorized HCC patients using risk scores, enabling dependable prognostic forecasts and identification of potential therapeutic targets for personalized treatment in HCC management. Future studies should explore integrating this model into clinical practice to improve patient outcomes.

## 1 Introduction

Liver cancer, particularly HCC, represents a significant global health issue, with rising morbidity and mortality rates in recent years. It is classified as the seventh most prevalent cancer and the second foremost cause of tumor-related fatalities globally (1, 2). Despite advancements in diagnostic and therapeutic techniques, liver cancer also presents a bleak outlook (3), with A general five-year survival percentage of nearly 21% (4). Current treatments like surgery, liver transplants, and systemic therapies are often limited by late-stage diagnosis and resistance to therapy (5, 6). Therefore, there is an urgent necessity for innovative prognostic models and therapeutic targets to enhance patient outcomes.

Telomere maintenance has emerged as a crucial factor in cancer progression and prognosis. During each cell division, telomeres, which are safeguarding caps at the ends of chromosomes, are reduced in length, causing cell aging or apoptosis when they become extremely short. However, cancer cells often activate mechanisms to maintain telomeres, such as telomerase activation or alternative lengthening of telomeres (ALT), to achieve replicative immortality (7–9). In liver cancer, telomerase reverse transcriptase (TERT) promoter mutations are frequently observed, indicating a pivotal role of telomere maintenance in hepatocarcinogenesis (10). Furthermore, telomere length and the telomerase activity have been linked to tumor aggressiveness and patient prognosis in various cancers, including liver cancer (11, 12).

In the context of liver cancer, aberrant TMM have been implicated in cancer metastasis and invasion (13). Telomere dysfunctions, often observed in precancerous lesions, are associated with genetic instability, driving the development of malignancy (14). Recent studies have emphasized the predictive potential of genes related to telomere maintenance in other types of cancer. For instance, a prognostic model based on genes associated with telomere function accurately forecasted survival outcomes in patients with bladder cancer, demonstrating the utility of such models in clinical practice (15). Similarly, in lung cancer, a gene signature linked to telomere maintenance correlated with patient survival and response to treatment, underscoring the relevance of telomere biology in cancer prognosis and treatment (16). Moreover, emerging evidence suggests that telomere dysfunction may influence therapeutic response and treatment outcomes in liver cancer, highlighting the prospect of TMM as an emerging predictive biomarker and a promising therapeutic target (17).

**Figure 1:**
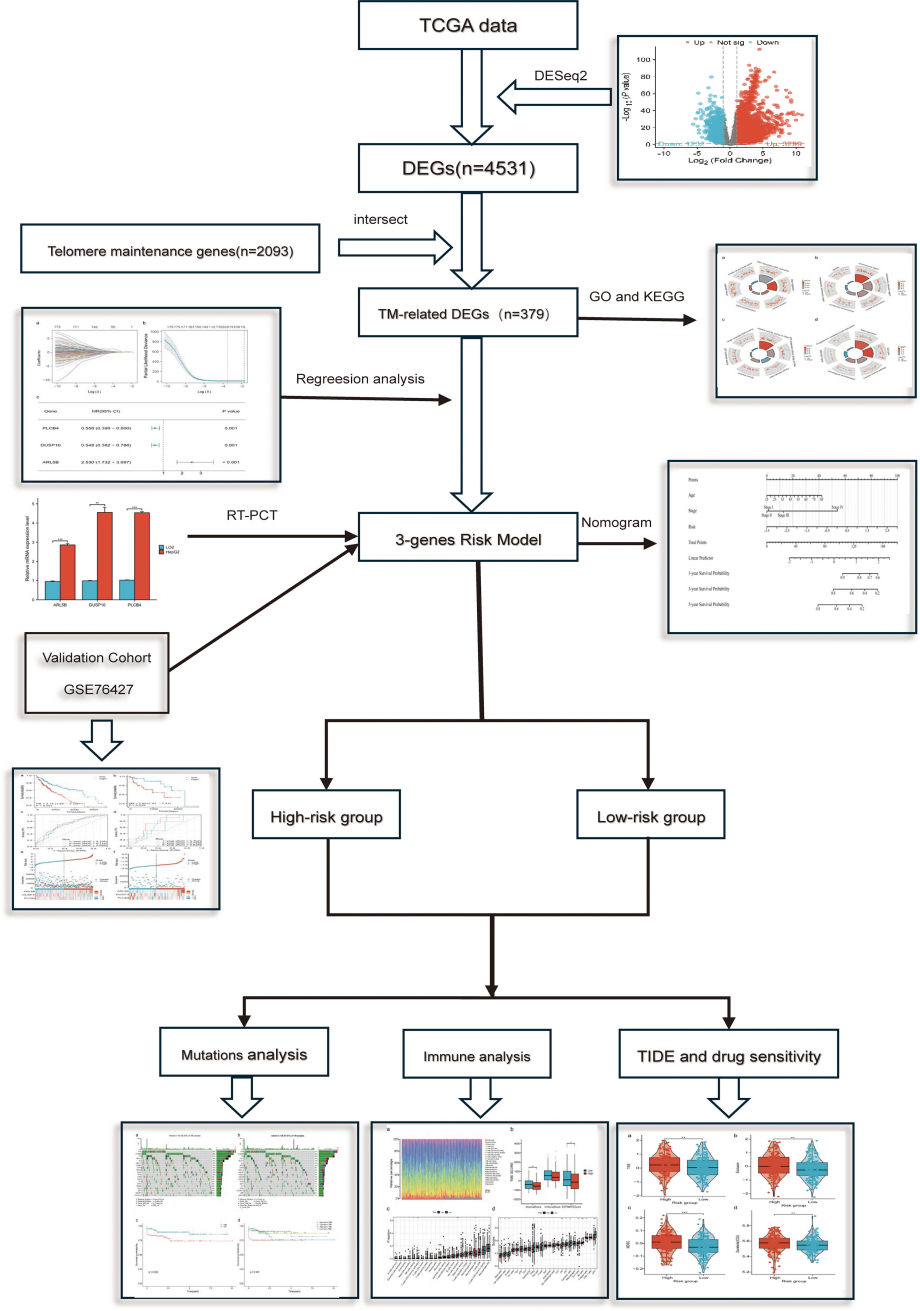
The process of this research.

Our findings demonstrate that the TMGs signature can effectively categorize liver cancer patients into two mortality risk categories with notable disparities in OS. The model also correlates with TMB, immune cell infiltration, and immunotherapy response, suggesting its potential usefulness in guiding personalized treatment strategies. By integrating clinical factors such as age and clinical stage, we developed a nomogram for individualized survival prediction, which could enhance clinical decision-making and accurately anticipate patient outcomes in tumor cases. The process of this research is shown in **Figure 1**.

## 2 Materials and methods

### 2.1 Data collection

Genes involved in telomere maintenance were collected from TelNet website (18). Gene expression and clinical information were acquired from the TCGA database. GSE76427 dataset was acquired through GEO data. Clinical data was preprocessed by eliminating samples without survival status. Additionally, patients with OS of less than 30 days were excluded due to the possibility of non-cancer-related causes of death (19, 20). We have included the web addresses for all databases in **Supplementary Table S1**.

### 2.2 Screening DEGs and enrichment analysis

DEGs between liver cancer tumors and normal samples in the TCGA dataset were identified using the DESeq2 package in R (adjusted p < 0.05 and |logFC| > 1) (21). DEGs were visualized using the package ggplot2. We utilized the clusterProfiler software to examine the pathways and functional enrichment through KEGG and GO.

### 2.3 Construction of predictive model

Univariate Cox regression was utilized to pinpoint genes linked to the prognosis of liver cancer concerning TMGs. Afterwards, LASSO was utilized to further enhance the selection of genes. Ultimately, multivariate Cox regression was conducted to pinpoint key prognostic genes (22, 23). A bilateral P-value < 0.05 was deemed significant. The R packages utilized included survival, rms, glmnet, eulerr, and ggplot2 (24). Multivariate Cox regression was employed to develop a predictive model. Ultimately, we calculated the risk score by multiplying the selected gene expression levels with the corresponding multivariate Cox regression analysis coefficients, subsequently categorizing HCC patients into two risk groups using the median risk score as the threshold value.

### 2.4 Evaluation of prognostic risk model

Kaplan-Meier (K-M) curves were used to compare differences in OS between two groups. The timeROC package was employed to assess the prognostic accuracy of the risk model using receiver operating characteristic (ROC) curves (25). The GEO dataset served as an independent validation cohort.

### 2.5 Building and adjustment of nomograms

To augment the predictive accuracy of the model and provide clinicians with a quantitative tool to estimate OS in patients with HCC, we constructed a nomogram by integrating the predictive model with clinical variables(tumor stage and age). Subsequently, the nomograms were evaluated using a calibration plot, which compares the predicted probability with the observed outcomes, and by calculating the concordance index.

### 2.6 Mutation analysis

TMB scores were computed using the maftools package, followed by a mutation analysis (26). HCC mutation files were acquired from TCGA-LIHC. Somatic mutations of risk groups were depicted using waterfall plots. Further comparisons were conducted to evaluate disparities in tumor mutation burden and prognostic results among the risk cohorts.

### 2.7 Immune correlation analysis

CIBERSORT, a deconvolution algorithm, evaluated the abundance of immune cells in each sample using gene expression data. Single-Sample GSEA (ssGSEA) was employed to compare immune cell populations across the various risk groups (27). We employed the estimation software to evaluate discrepancies in stromal, immune, and total scores among these groups. The correlations between TMGs and liver cancer immune cells were illustrated using ssGSEA and CIBERSORT algorithm.

### 2.8 Analysis of the immunotherapy response and drug sensitivity

TIDE score is extensively employed to assess the efficacy of tumor immunological therapy. We employed the TIDE platform to determine the responsiveness of immunologic therapy and illustrated the outcomes using the TIDE index. The “pRRophetic” R package is utilized to indicate therapeutic sensitivity (28).

### 2.9 Cell culture and real-time PCR

The normal liver LO2 cells (iCell-h054, iCell Bioscience Inc., China) and HCC HepG2 cells (iCell-h092, iCell Bioscience Inc., China) were cultured in RPMI 1640 medium (Cytiva, USA) with 10% FBS and 1% penicillin/streptomycin under standard environment of 37°C and 5% CO_2_.

TRIpure reagent was used to extract total RNA according to the manufacturer’s instructions. cDNA was synthesized using the EntiLink™ 1st Strand cDNA Synthesis Super Mix (ELK Biotechnology, Wuhan, China). RT-PCR was performed utilizing the QuantStudio 6 Flex System PCR provided by Life technologies Company. The primer sequences used were shown in **Supplementary Table S2**.

### 2.10 Statistical analysis

All analyses were conducted using R statistical software (version 4.2.1). The log rank test were employed to compare the OS between 2 different populations. The Wilcoxon test was employed to assess continuous parameters between two groups. Correlation analysis was conducted utilizing the Spearman method. P values < 0.05 indicated statistically significant.

## 3 Results

### 3.1 Identification and gene enrichment in liver cancer

**Figure 2:**
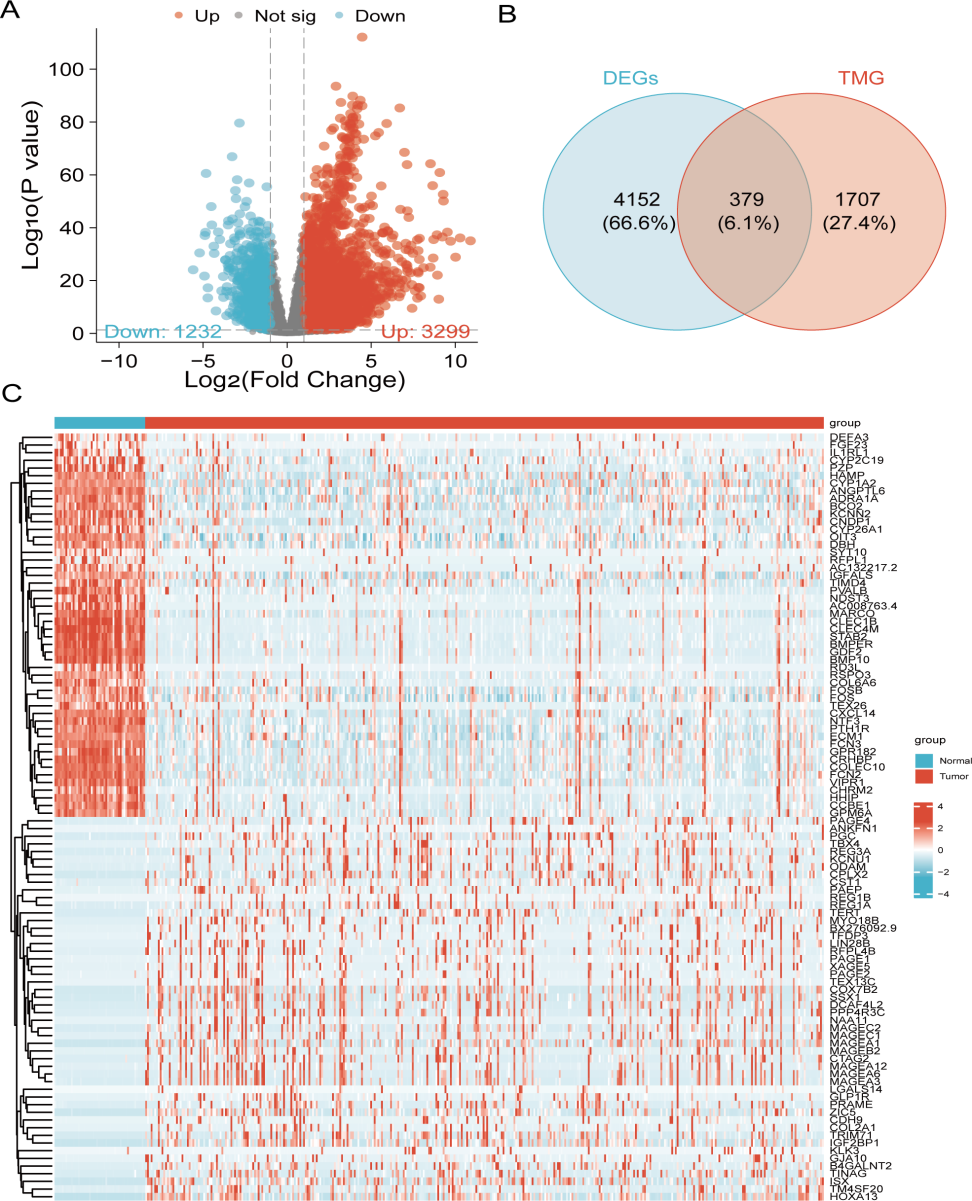
Identification of liver cancer-associated DEGs involved in telomere maintenance. **(A)** Volcano plot of 4,531 genes with differential expression. **(B)** Venn diagram illustrating the overlap between DEGs and TMGs in liver cancer patients. **(C)** Heatmap displaying the top 50 upregulated and downregulated genes.

Significant differential expression in malignant tissues, as opposed to on-cancerous tissues, was demonstrated by 19,962 genes through the volcano plot analysis. Among these, we identified 4,531 that were significant DEGs, including 3,299 upregulated and 1,232 downregulated genes (**Figure 2A**). By intersecting the DEGs with a set of 2,093 TMGs, we discovered 379 overlapping genes (**Figure 2B**). A heatmap was generated by selecting the 50 most distinct points from the green and red regions of the volcano plot (**Figure 2C**).

### 3.2 Functional enrichment analysis of DEGs

**Figure 3:**
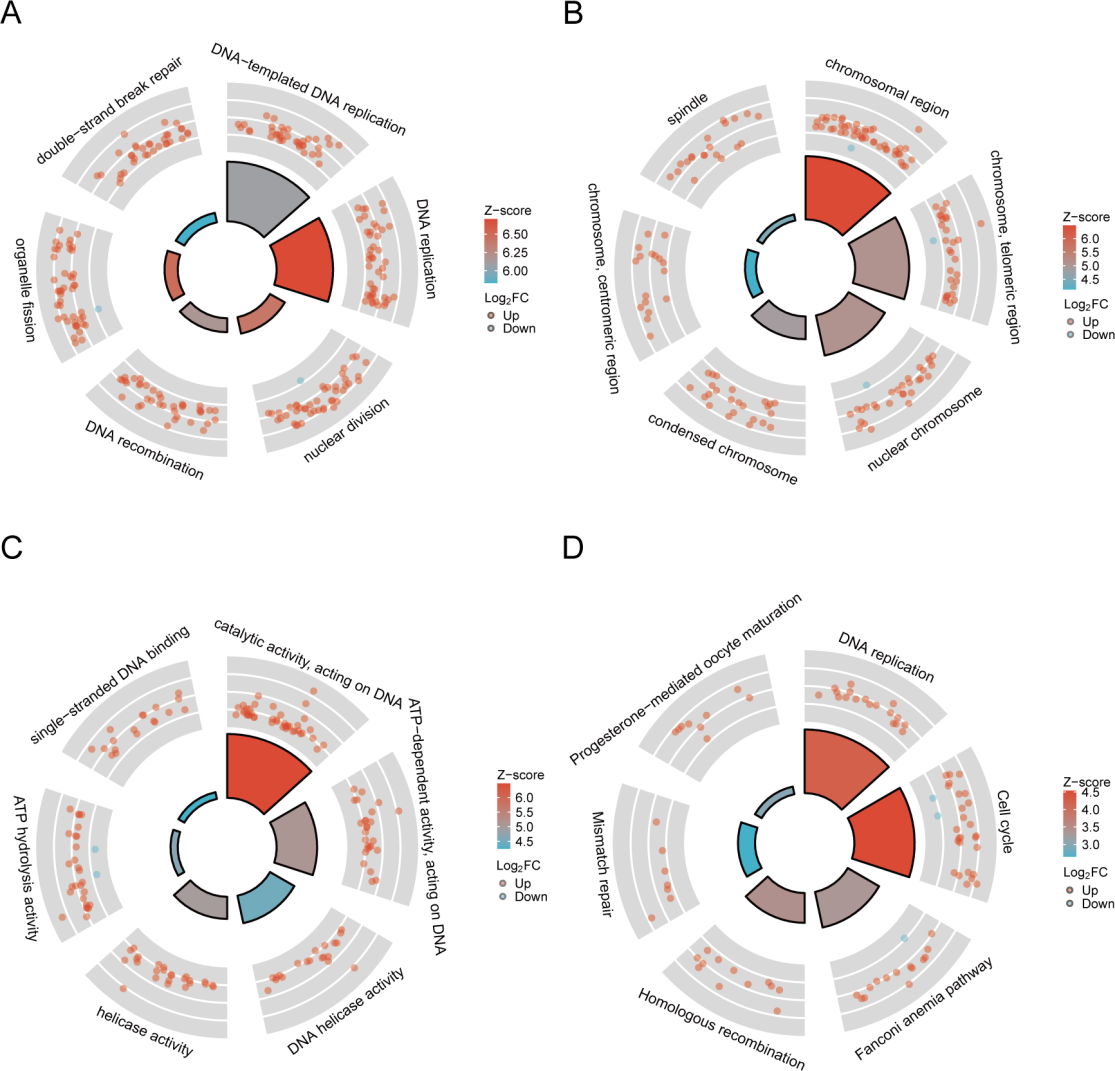
The outcomes of functional enrichment analysis. **(A–C)** GO analysis of the TMGs. **(D)** KEGG pathway enrichment analysis of the TMGs.

We conducted GO and KEGG analysis to gain functional assignment and categorization of the DEGs associated with telomere maintenance. GO enrichment analysis of the 379 telomere maintenance-associated DEGs revealed the following: In terms of biological process, The regulation of organelle fission, DNA replication, and nuclear division were primarily related to the DEGs. In terms of cellular component, The processes related to chromosome areas, telomeric chromosome regions, and nuclear chromatin were considerably enriched with differentially expressed genes. Regarding molecular functions, the differentially expressed genes were associated with enzymatic activity, roles as DNA-binding activators, and transcription factor activity specific to RNA polymerase II. (**Figures 3A–C**). It was revealed by KEGG analysis that the pathways were predominantly connected with the cellular cycle, senescence of cells, and DNA replication (**Figure 3D**).

### 3.3 Acquisition of three prognostically relevant differential genes and building the model

**Figure 4:**
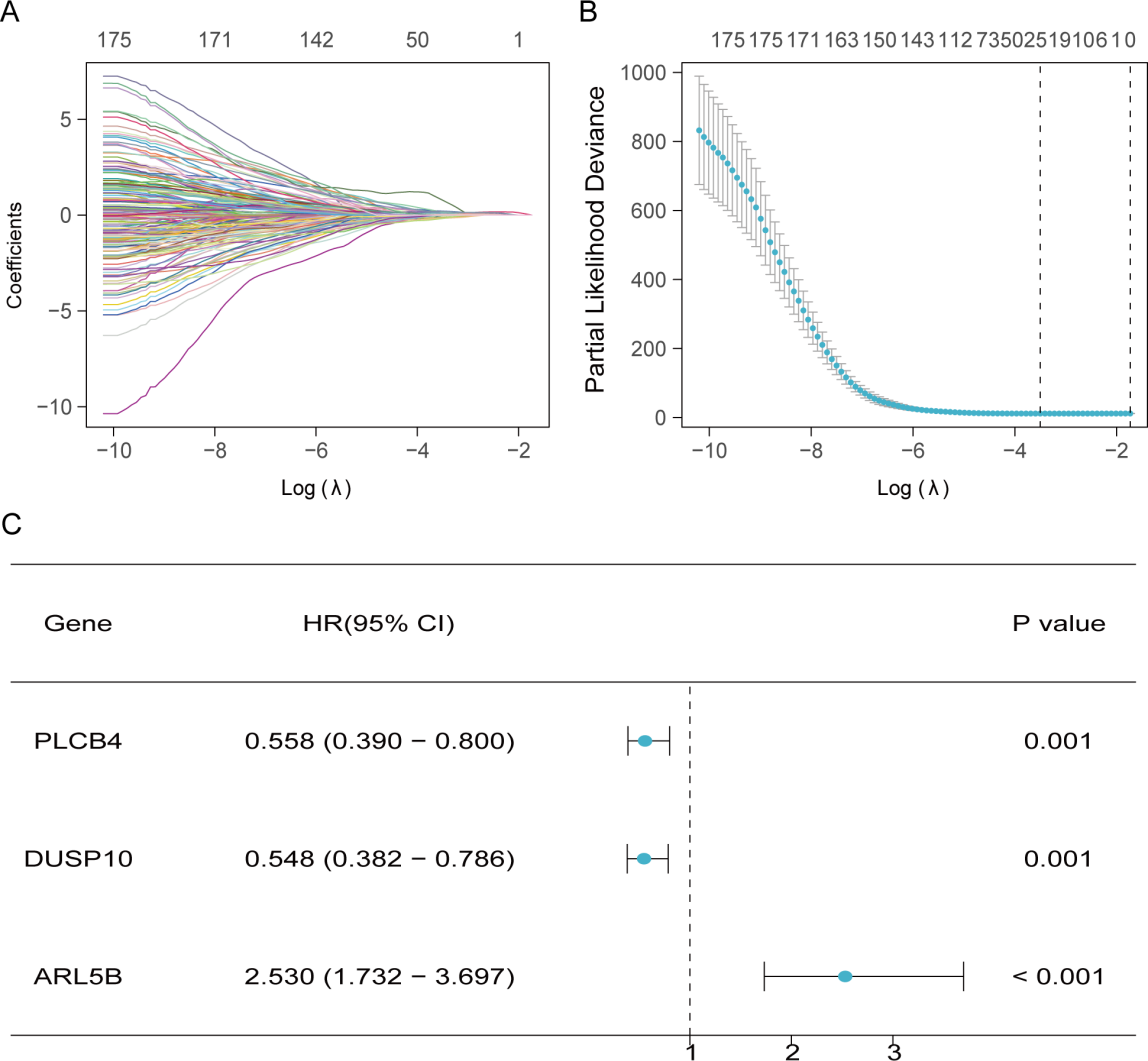
Development of forecasting model related to TMGs. **(A, B)** Twenty-two prognostic-associated TMGs were discerned through LASSO analysis. **(C)** The forest plot of 3 prognostically relevant TMGs.

To identify prognostic-related TMGs in liver cancer, univariate analyses with the Cox regression model was performed on the 379 overlapping genes, resulting in the identification of 175 candidate genes. Using Lasso algorithms, we screened out 22 candidate genes (**Figures 4A, B**). Finally, 3 core genes (PLCB4, DUSP10, ARL5B) were recognized as independent predictors through the multivariate analyses with the Cox regression model and these were employed to formulate the prediction model.

**Figure 5:**
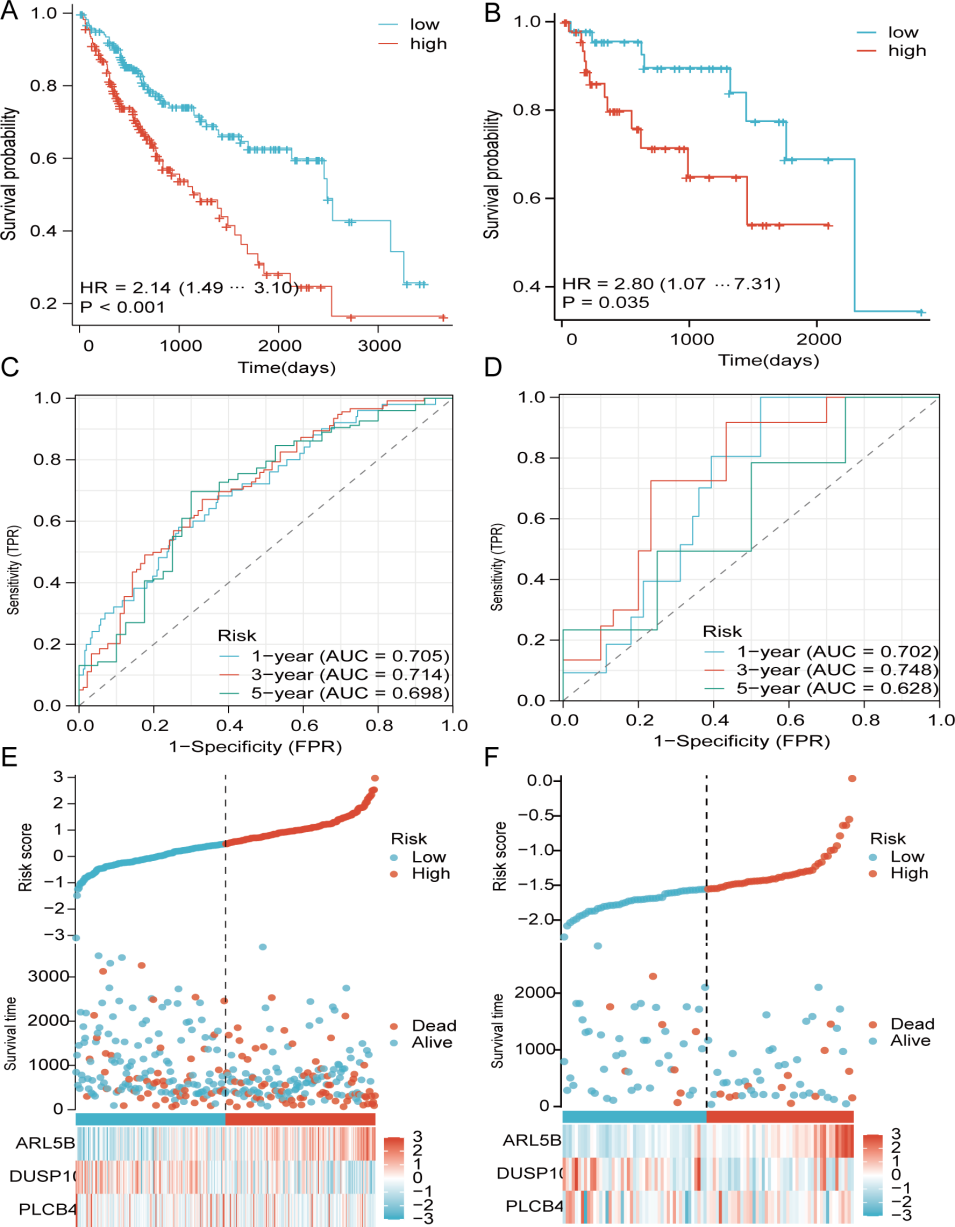
Dissemination of survival outcomes and risk assessments for TCGA and GEO. **(A, B)** survival analysis of TCGA and GEO. **(C, D)** ROC curves analysis. **(E, F)** Heatmap images of alterations in TCGA and GEO gene expression.

Additionally, we computed the risk score for each TCGA patient based on expression levels of the 3 genes and the coefficients of multivariate Cox regression analysis (risk score = -0.5826967*PLCB4+ -0.60188072*DUSP10 + 0.92837317*ARL5B). Patients from TCGA and GEO were ranked by risk score and divided into high- and low-score individuals on basis of the optimal truncation value of risk score. In the modeling cohorts, the OS curves revealed that the high-score individuals were significantly lower than others (**Figures 5A, E**). Moreover, the AUC (Area under the Curve) serves as an indicator for assessing the efficacy of survival prediction models, with elevated values signifying enhanced model precision in differentiating various survival outcomes, and the ROC curve illustrates the AUC in **Figure 5C**. Likewise, the low-risk cohorts in the validation groups exhibited a more favorable prognosis, as evidenced by K-M curves indicating that patients with reduced risk scores experienced an improved survival outcome. Furthermore, ROC analysis of the GEO cohort validates that the risk model possesses an equally robust predictive capability as TCGA(**Figures 5B, D, F**).

### 3.4 Plotting of a nomogram

**Figure 6:**
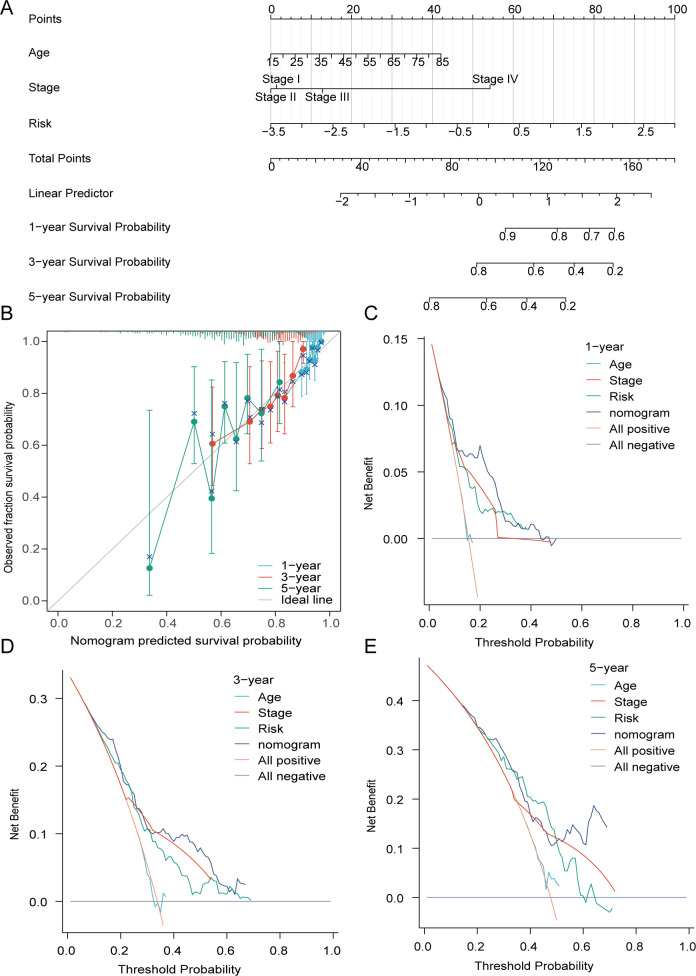
The formulation of the nomogram was carried out. **(A)** A prognostic nomogram founded on risk, disease stage, and age. **(B)** The alignment between the anticipated survival probability and the actual survival proportion was strongly demonstrated by the calibration curve at 1-year and 3-year. **(C–E)** DCA of prognostic nomogram, risk, age, and disease stage in forecasting 1-, 3-, and 5-year survival rates.

From these studies, we merged the risk with clinically available age and disease stage to construct an enhanced nomogram (**Figure 6A**). The calibration plot indicates that the observed values closely align with the predicted values. Based on these findings, and through decision curve analysis (DCA), the risk scores, in conjunction with various clinical characteristics, exhibit clinical efficacy (**Figures 6B–E**).

### 3.5 Tumor mutations were analyzed

**Figure 7:**
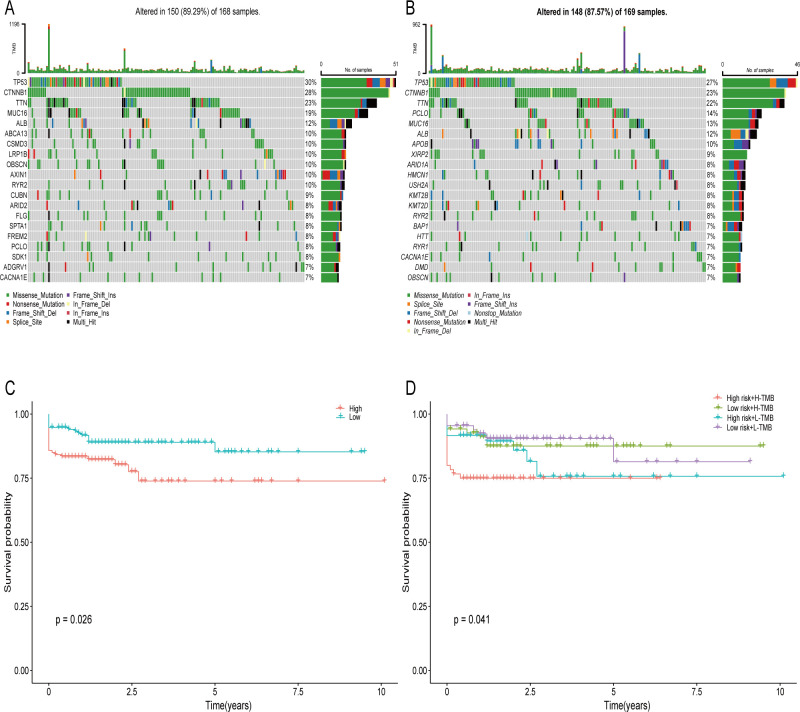
Somatic Mutation Comparison. **(A)** The twenty most commonly mutated genes in high-score groups. **(B)** The twenty most commonly mutated genes in low-score populations. **(C, D)** Prognostic evaluation of elevated and diminished tumor mutation load in populations at risk groups.

The examination of tumor mutations revealed that the primary forms of mutations were consistent across both two groups, although there were variances in mutation forms between two groups (**Figures 7A, B**). Patients exhibiting a high mutation load experienced a diminished survival rate in contrast to those with a low mutation load. Furthermore, the survival rate was markedly reduced in patients from both the high-risk and high mutation load groups in comparison to those in the low risk and low mutation load groups (**Figures 7C, D**).

### 3.6 Association of the tumor immune microenvironment with TMGs-related risk score

**Figure 8:**
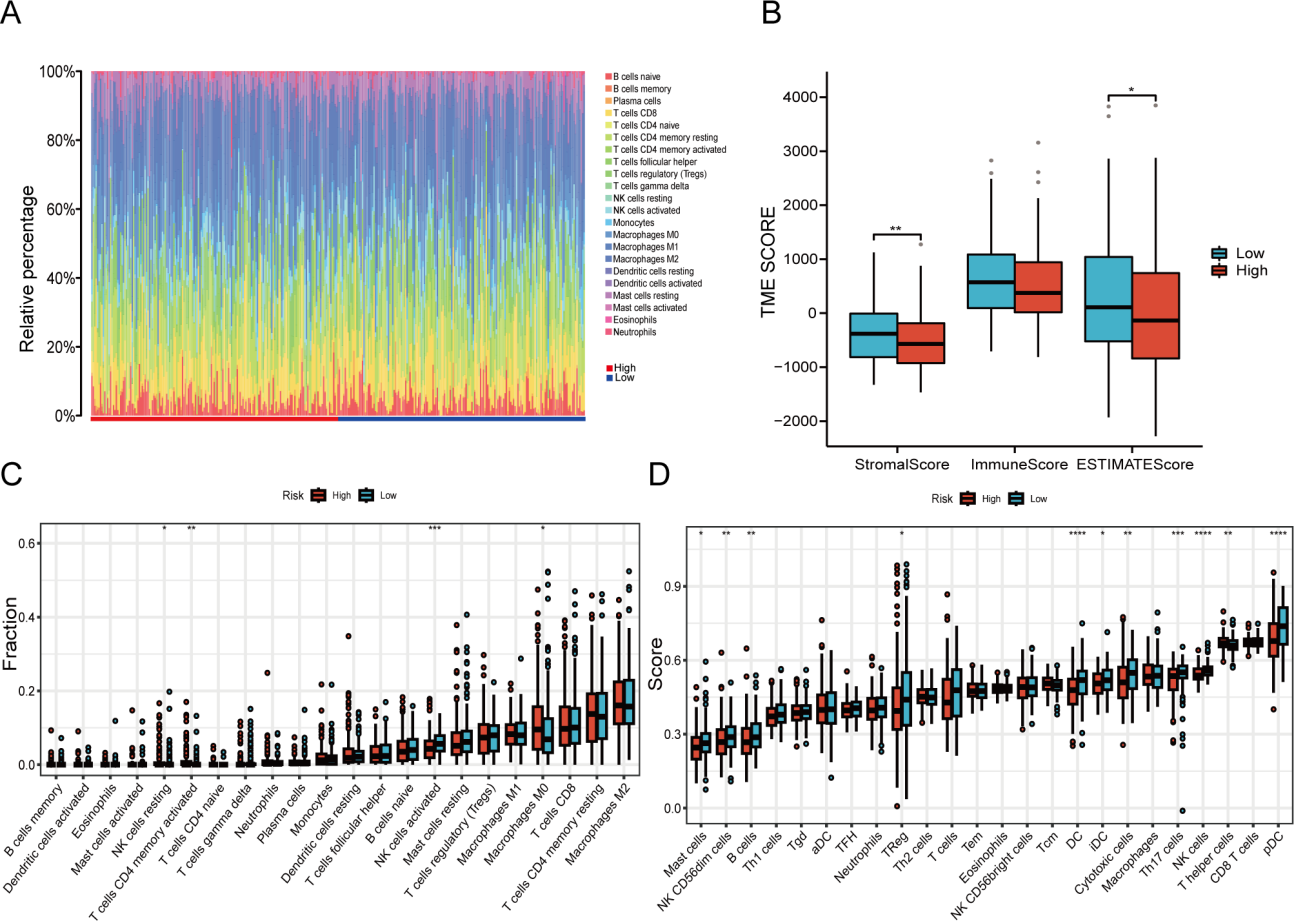
The TME characteristics vary among different risk groups. **(A, C)** An immune deconvolution algorithm (CIBERSORT) to deduce the infiltration of immune cells in two cohorts. **(B)** The TME scores derived from the ESTIMATE algorithm are evaluated for inconsistencies. **(D)** Discrepancy analysis of immune-associated score among risk groups based on ssGSEA algorithm (*P < 0.05, **P < 0.01, ***P < 0.001, ****P < 0.0001).

**Figure 9:**
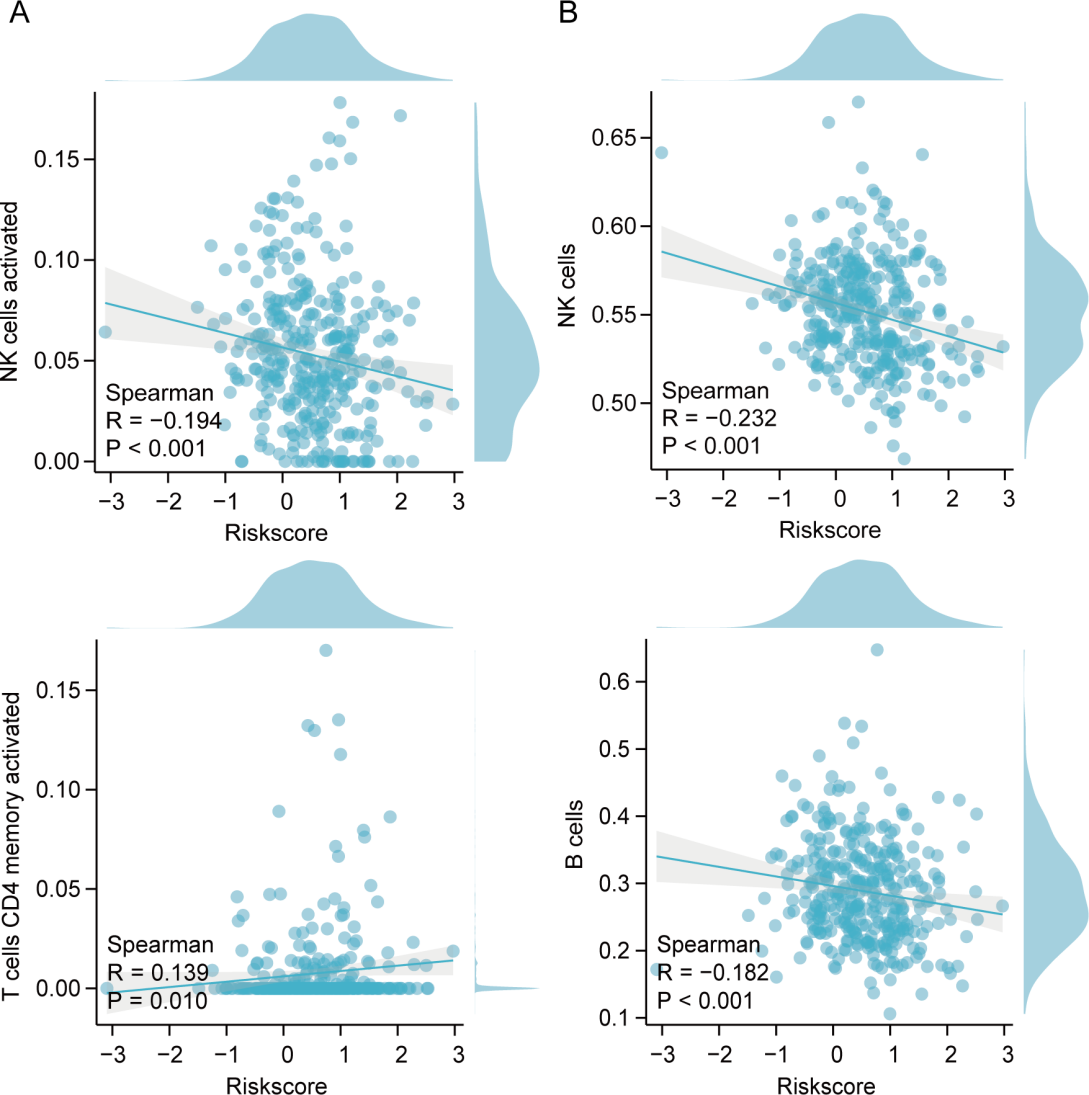
Spearman’s correlation between immunocytes and risk index. **(A)** Correlation of immune cells and risk model using CIBERSORT. **(B)** Correlation of immune cells and risk models using ssGSEA.

CIBERSORT analysis unveiled notable differences in immune cell infiltration, especially among CD4+ lymphocytes, natural killer (NK) cells, and macrophages between the two groups (**Figures 8A, C**). Meaningful distinctions in the infiltration properties of Th17 cells, NK cells, and other types of immunocytes between the two groups were demonstrated by the ssGSEA algorithm (**Figure 8D**). The estimation algorithm revealed that the overall score of the high-risk cohort were markedly diminished (**Figure 8B**). Associations with B cells, CD4+ lymphocytes, and NK cells were demonstrated by the risk model (**Figures 9A, B**).

### 3.7 The prediction of tumor reaction to immunological treatment

**Figure 10:**
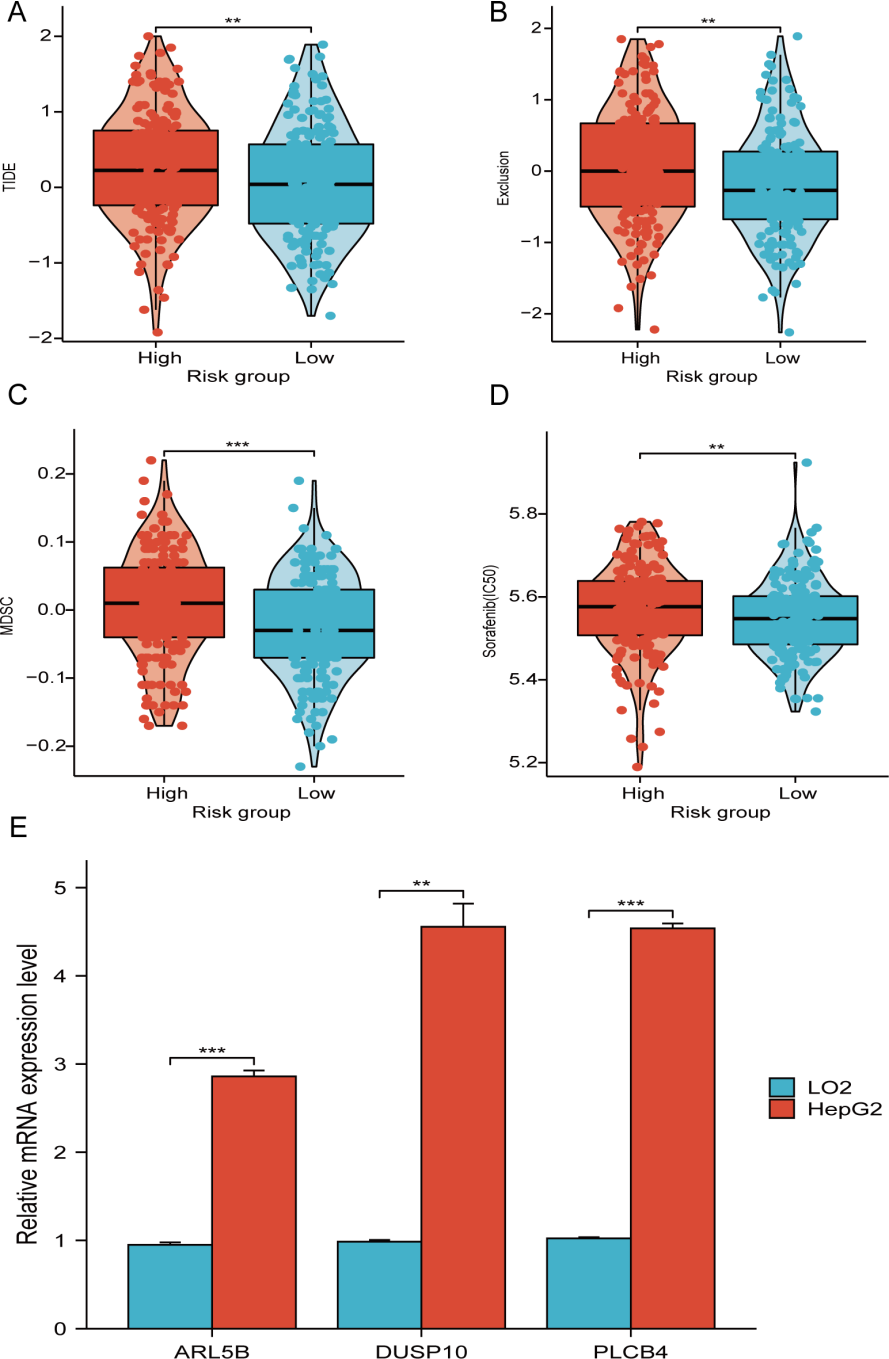
TIDE score and therapeutic sensitivity of medications. **(A–C)** Correlation analysis between TIDE score and risk score. **(D)** IC50 values of the targeted medication sorafenib in risk groups. **(E)** ARL5B, DUSP10, and PLCB4 mRNA expression in LO2 and HepG2 cells. **P < 0.01, ***P < 0.001.

Marked elevations in the TIDE, MDSC, and Exclusion scores were noted in the high-score individuals compared to the other individuals, indicating an elevated likelihood of immune evasion among high-risk patients. (**Figures 10A–C**). Consequently, Less benefit from immune checkpoint inhibition therapy is experienced by individuals in the high risk category. Furthermore, an elevated value of half maximal inhibitory concentration (IC50) for sorafenib was observed in high-risk individuals compared to others, signifying a reduced sensitivity to sorafenib among the high-risk individuals (**Figure 10D**).

### 3.8 Relevance of hub gene expression level

RT-PCR analysis reveals significant differences in the mRNA expression of three genes associated with telomere maintenance, which are crucial to our prognostic risk model, when comparing HepG2 cells to normal liver cells. In particular, the mRNA levels of PLCB4, DUSP10, and ARL5B are notably higher in HepG2 cells than in LO2 cells (**Figure 10E**). These results suggest that the altered expression of these genes may play a role in the development and progression of hepatocellular carcinoma.

## 4 Discussion

Liver malignancy, including HCC, is one of the most widespread and fatal cancers globally, representing a significant health burden (29–31). Our investigation focuses on constructing a predictive risk model grounded in TMGs, utilizing transcriptomic and clinical data from liver cancer patients. This approach holds promise for enhancing the diagnosis and treatment of liver cancer by identifying key molecular mechanisms involved in its progression.

TMM empowers cancer cells with the ability to proliferate continuously. Targeting TMM is a well-established approach in cancer treatment (11). A prior study demonstrated that TMM had significant prognosis value in pancreatic adenocarcinoma, and head and neck squamous cell carcinoma (32). Through our research, a prognostic model related to TMGs was established, leading to the successful classification of patients into two groups with distinct survival outcomes. ROC curve analysis demonstrated that the 1-year and 3-year AUC values were superior, further validating the predictive power of our model compared to similar prognostic models in the study by Fan M et al (33). Importantly, the present study identified 3 core TMGs (PLCB4, DUSP10, and ARL5B). These genes exhibited significant correlations with OS in HCC patients.

ARL5B, a constituent of the ADP ribosylation factor-like family, is part of the RAS superfamily (34). Studies have shown that heightened ARL5B expression promoted the movement of lysosomes, leading to their dispersal and accumulation at the outer edge of the cell (35). Prior research revealed that impaired lysosomal exocytosis led to reduced malignant cell invasion (36). Previous research has indicated that ARL5B augmented the translocation and infiltration of breast cancer cells, suggesting its oncogenic function in breast cancer (35). Furthermore, suppression of ARL5B resulted in a decrease in the scattering of lysosomes and subsequently led to a reduction in cell invasion in prostate cancer (37). The PLCB4 gene, which encodes the PLCβ4 protein, is a sizable gene spanning 412 kb (38). Changes in PLCB4 expression are associated with a decline in survival rates in patients with solid tumors, including mesothelioma, colorectal cancer, and gastrointestinal tumors (39, 40). PLCB4 hypomethylation has been linked to the development of HCC and the survival of liver cancer without recurrence (41, 42). Within the human genome sequence of Dual-specificity phosphatase 10 (DUSP10), two transcripts have been recognized, with the longer transcript being extensively expressed across diverse human tissues, including the liver (43). DUSP10 is associated with inflammation, cytokine release, cellular growth, cellular movement, viability, and programmed cell death (44, 45). Several research have found elevated levels of DUSP10 messenger RNA in cancerous tissues, suggesting a cancer-promoting role. Conversely, lowered expression of DUSP10 seems to correlate with migration and spread in HCC (43, 46, 47). Our research discovered diminished DUSP10 expression in the high-risk cohort.

TMB is gaining recognition as diagnostic markers to anticipate the prospective benefit of immune checkpoint inhibitors (ICIs) therapy (25, 26). An increased number of mutations leads to an amplification in neoantigen and immunogenic recognition, thereby promoting T-cell responses stimulated against tumor cells. Consequently, an elevated TMB may indicate an intensified responsiveness to immunotherapy (48). An inferior prognosis was noted in patients with high mutation load compared to those with low mutation load (49). Mutations in the TERT promoter demonstrated the highest frequency, occurring in 60% of HCC patients, suggesting that genes associated with telomere maintenance may be crucial in the development of HCC (50, 51). The findings of tumor mutation load analysis demonstrated that the prognosis of tumor mutation load in low-risk individuals was markedly superior to that in others. This discovery underscores the potential of telomere preservation genes as biomarkers for predicting the effectiveness of ICIs in liver cancer.

The immune microenvironment of hepatocellular carcinoma is crucial in cancer progression and patient prognosis (52–54). The model based on the aforementioned trio of telomere maintenance genes has the capability to forecast patients’ prognosis. Additionally, it can be utilized to anticipate a patient’s response to immunotherapy. This research employed ESTIMATE, ssGSEA, and CIBERSORT software to analyze the immune cells infiltration in HCC tissues, uncovering noteworthy disparities among risk groups. Notably, we noted pronounced differences in the abundance of CD4+ lymphocytes, NK cells, and other types of immunocytes, suggesting that telomere maintenance not only contributes to the development of TME but also potentially regulates the activity of immunocytes. The TIDE index was diminished in the low-risk individuals relative to others, Indicating a more favorable response to immunologic treatment. These findings indicate that telomere maintenance genes could impact immune responses to HCC therapy by modulating the efficacy of immunocytes. Telomere maintenance gene might serve as a novel target for liver cancer treatment. Sorafenib, a multitargeted tyrosine kinase inhibitor (55), emerged as the initial therapy to exhibit effectiveness in patients with advanced hepatocellular carcinoma, significantly extending the overall median survival (56). In the current investigation, the IC50 of sorafenib was diminished in the low-risk cohorts. Consequently, drawing upon the telomere maintenance-associated gene profile, we propose that liver cancer patients with diminished risk may exhibit increased susceptibility to targeted therapy.

While this research is clinically important for evaluating prognosis and treatment options for liver cancer patients, it has several limitations. Firstly, Given that our research was exclusively validated through bioinformatics and PCR conducted in cell cultures, it is essential to pursue further validation in future prospective studies and animal trials. Secondly, the sample size of liver cancer patients included in the study was limited, potentially introducing bias into the results. Thirdly, because there is no comprehensive clinical validation, these prognostic models need further testing in large-scale prospective clinical studies to confirm their practical use. Additionally, using multiple datasets from various sources could introduce batch effects, impacting the robustness and reproducibility of the findings. Future research should aim to address these limitations by incorporating experimental validation, expanding sample sizes, and applying rigorous methods to minimize batch effects.

## 5 Conclusion

To summarize, This study formulated a predictive risk model employing TMGs, which effectively classified patients into high- and low-risk categories. This stratification showcased notable disparities in OS and displayed strong predictive capability. Moreover, substantial variations were identified among risk groups regarding tumor mutation load, the prevalence of immunocytes, and the activity of immune pathways. The TIDE algorithm highlighted distinct reactions to immune therapies and sensitivities to medications between the two groups. These conclusions illuminate the potential of TMGs as diagnostic markers and treatment targets in hepatic cancer. Future studies should strive to validate these findings in larger, independent cohorts and explore the underlying biological mechanisms through experimental research.

## Data Availability

The original contributions presented in the study are included in the article/**Supplementary Material**. Further inquiries can be directed to the corresponding author/s.
