# Supplementary material for: Develop a prognostic and drug therapy efficacy prediction model for hepatocellular carcinoma based on telomere maintenance-associated genes
Source: Front Oncol. 2025 Feb 14;15:1544173. doi: 10.3389/fonc.2025.1544173 (PMC11867940; doi:10.3389/fonc.2025.1544173)
Supplement: Supplementary file 1 [file Table1.docx]

**Supplementary Table S1**

| Name | Web address |
| --- | --- |
| The Cancer Genome Atlas (TCGA) | https://portal.gdc.cancer.gov/ |
| Gene Expression Omnibus (GEO) | https://www.ncbi.nlm.nih.gov/geo/ |
| TelNet website | https://www.cancertelsys.org/telnet/ |
