# Supplementary material for: Develop a prognostic and drug therapy efficacy prediction model for hepatocellular carcinoma based on telomere maintenance-associated genes
Source: Front Oncol. 2025 Feb 14;15:1544173. doi: 10.3389/fonc.2025.1544173 (PMC11867940; doi:10.3389/fonc.2025.1544173)
Supplement: Supplementary file 2 [file Table2.docx]

**Supplementary Table S2.** Primer sequences used in this study

| Gene | Sequence |
| --- | --- |
| *PLCB4* | Forward 5’- -3’ GCCACTGCAGACACAAACTATC |
|  | Reverse 5’- -3’ GCCACTGCAGACACAAACTATC |
| *DUSP10* | Forward 5’- -3’ CTGACATCGAGAACGCTGAGC |
|  | Reverse 5’- -3’ GTGGTGACGTTGATGACGTAGC |
| *ARL5B* | Forward 5’- -3’ GAAGTGGTTCATACTTCTCCAACC  Reverse 5’- -3’ AATGCTATCAACAACAAGAATGATG |
| *ACTIN* | Forward 5’- -3’ GTCCACCGCAAATGCTTCTA  Reverse 5’- -3’ TGCTGTCACCTTCACCGTTC |
